# Pre-Treatment with Dacarbazine Sensitizes B16 Melanoma to CAR T Cell Therapy in Syngeneic Mouse Model

**DOI:** 10.3390/ijms27010189

**Published:** 2025-12-24

**Authors:** Egor A. Emelianov, Elizaveta R. Naberezhnaya, Andrey S. Logvinov, Valeria M. Stepanova, Aleksandr S. Chernov, Yuliana A. Mokrushina, Diana M. Malabuiok, Dmitry E. Pershin, Ekaterina A. Malakhova, Elena A. Kulakovskaya, Tatiana N. Prokofeva, Victor V. Tatarskiy, Elena I. Shramova, Sergey M. Deyev, Alexander G. Gabibov, Nikolay E. Kushlinskii, Yury P. Rubtsov, Dmitry V. Volkov

**Affiliations:** 1 Shemyakin-Ovchinnikov Institute of Bioorganic Chemistry, Russian Academy of Sciences, 117997 Moscow, Russia; egor2001nn@gmail.com (E.A.E.); elizaveta2001@gmail.com (E.R.N.); globulin992@gmail.com (A.S.L.); ukrainskaya49@gmail.com (V.M.S.); alexandrchernov1984@gmail.com (A.S.C.); yuliana256@mail.ru (Y.A.M.); malabuiokdiana@gmail.com (D.M.M.); absent_seva@list.ru (T.N.P.); shramova.e.i@gmail.com (E.I.S.); biomem@mail.ru (S.M.D.); gabibov@mx.ibch.ru (A.G.G.); 2 Dmitry Rogachev National Medical Research Center of Pediatric Hematology, Oncology and Immunology, 117997 Moscow, Russia; serotonin16@mail.ru (D.E.P.); mallahovka@yandex.ru (E.A.M.); alenakulakovskaya@gmail.com (E.A.K.); 3 Laboratory of Molecular Oncobiology, Institute of Gene Biology, 34/5 Vavilova Street, 119334 Moscow, Russia; tatarskii@gmail.com; 4 N.N. Blokhin National Medical Research Center of Oncology, Ministry of Health of the Russian Federation, 115522 Moscow, Russia; kne3108@gmail.com

**Keywords:** B16 melanoma, murine CAR T cells, dacarbazine, tumor microenvironment, syngeneic mouse model, combined melanoma therapy

## Abstract

Adoptive cell therapy (ACT) with T cells modified with a chimeric antigen receptor (CAR T cells) has dramatically improved outcomes in hematologic cancers. However, its efficacy in solid tumors, such as melanoma, is hampered by several factors. These include heterogeneous expression of tumor-associated antigens (TAA) and an immunosuppressive, profibrotic tumor microenvironment (TME), which restricts cytotoxic CAR T cells trafficking into the tumor, as well as their persistence and cytolytic activity. As a result, responses to CAR T cell monotherapy in melanoma and other solid tumors are typically weak, transient or even absent. Emerging evidence suggests that combining traditional chemotherapy with CAR T cell therapy can enhance the antitumor activity of CAR T cells in solid malignancies. Partial tumor cell killing by chemotherapy improves access to TAA and disrupts the TME by affecting the global structure of the tumor tissue. Here, we developed an immunocompetent syngeneic B16 melanoma mouse model to test a combination of classical dacarbazine (DTIC) chemotherapy with ACT with murine CAR T cells. B16-F10 (next as B16) melanoma cells were modified to express a human/murine hybrid epidermal growth factor receptor (EGFR) recognized by a murine CAR bearing a single-chain variable fragment (scFv) derived from cetuximab, an anti-EGFR monoclonal antibody approved for the treatment of colorectal and certain other solid tumors. Prior to CAR T cells administration, cyclophosphamide (CPA) pre-conditioning was used. We demonstrated that DTIC therapy followed by infusion of murine CAR T cells targeting the human/murine hybrid EGFR (EGFR mCAR T cells) provided superior tumor control and prolonged survival compared to monotherapy with either DTIC or EGFR mCAR T cells alone. These findings support the potential feasibility of a combined therapeutic strategy for human melanoma involving DTIC treatment followed by EGFR CAR T cells infusion after CPA pre-conditioning.

## 1. Introduction

Therapy with T cells modified with a chimeric antigen receptor (CAR T cells) has revolutionized the treatment of hematologic malignancies, inducing durable remissions and, in some cases, complete cures [1,2]. In contrast, the efficacy of CAR T cells in solid tumors, including melanoma, remains insufficient. Objective response rates rarely exceed 10–25%, and tumor control is typically short-lived [3,4,5,6]. Multiple barriers within the tumor microenvironment (TME) restrict CAR T cells activity in melanoma. Dense stroma and abnormal vasculature form physical obstacles to CAR T cells infiltration [7,8], while inhibitory signaling pathways, including PD-1/PD-L1, TIM-3/galectin-9, and TIGIT/CD155, suppress effector function [9,10]. In addition, the accumulation of regulatory T cells (Treg) and myeloid-derived suppressor cells (MDSC) within the TME contributes to immunosuppression and CAR T cells dysfunction [11]. Collectively, these factors create an exhausting TME that compromises adoptive cell therapy (ACT), such as CAR T cell therapy.

Chemotherapy, once considered solely as tumor lytic, is now recognized for its ability to affect the TME and enhance antitumor immunity. Several chemotherapeutic agents can promote immunogenic cell death (ICD), characterized by the release of danger-associated molecular patterns such as ATP and HMGB1 and the exposure of calreticulin, which activate dendritic cells and enhance T cell priming [12]. Concurrently, chemotherapy can reduce populations of Treg and MDSC [13], upregulate chemokines to improve T cells infiltration [14], and increase the expression of tumor-associated antigens (TAA) and major histocompatibility complex class I molecules, making malignant cells more recognizable to immune effectors, including CAR T cells [15]. Dacarbazine (DTIC), a chemotherapeutic widely used in melanoma, has been shown to induce ICD within 6–24 h and upregulate NKG2D ligands, thereby sensitizing tumors to PD-1 blockade [16]. Cyclophosphamide (CPA), when administered at lymphodepleting doses, complements this by transiently depleting immunosuppressive cells and inducing the release of homeostatic cytokines such as interleukin-7 (IL-7) and IL-15 [17,18].

In melanoma specifically, preclinical studies have demonstrated the potential of combining CAR T cell therapy with chemotherapeutic agents. Melanoma-associated targets such as GD2, CSPG4, and B7-H3 have been utilized to engineer CAR T cells with antitumor activity both in vitro and in vivo [19,20,21]. When combined with chemotherapy—either as lymphodepleting pre-conditioning or as concurrent treatment—these CAR T cells often exhibit improved tumor infiltration, persistence, and cytotoxic function. Agents such as CPA and fludarabine have been employed to enhance T cell engraftment [22], while others, like temozolomide, may modulate the TME to favor immune activity [23]. Nevertheless, identifying effective drug partners for CAR T cells, optimizing dosing schedules and therapeutic windows, and managing toxicity remain critical challenges in developing combinatorial therapies for solid tumors.

Here, we demonstrate for the first time that, in a syngeneic B16 melanoma model engineered to express a human/murine hybrid epidermal growth factor receptor (EGFR), a fractional-dose regimen of DTIC effectively complements the subsequent adoptive transfer of murine CAR T cells specific to the hybrid EGFR. This combination therapy results in both sustained inhibition of tumor growth and prolonged survival in treated mice. Overall, our findings support the therapeutic potential of combinatorial strategies, which are increasingly regarded as a critical next step in advancing cancer treatment.

## 2. Results

### 2.1. Generation of Human/Murine Hybrid EGFR and EGFR-Specific Murine CAR

**Figure 1:**
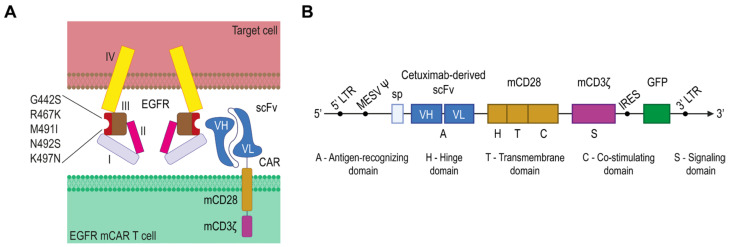
Design of human/murine hybrid epidermal growth factor receptor (EGFR) and EGFR murine chimeric antigen receptor (CAR). (**A**) Schematic of the human/murine hybrid EGFR with cetuximab-binding substitutions (G442S, R467K, M491I, N492S, K497N) and its engagement by a cetuximab-derived single-chain variable fragment (scFv) on mCAR T cells. (**B**) The retroviral vector encoding the EGFR-specific murine CAR and green fluorescent protein (GFP); I—domain I; II—domain II; III—domain III; IV—domain IV.

EGFR is a well-established marker in multiple cancers, including melanoma [24]. Given its prominent role in tumor biology, EGFR has become a compelling target for CAR T cell therapy. Human EGFR expressed on various tumor types was selected as the target. To create a humanized variant of murine EGFR recognizable by cetuximab, several amino acid substitutions were introduced (G442S, R467K, M491I, N492S, K497N) (Figure 1A) [25,26]. Cetuximab, a monoclonal antibody that inhibits EGFR, is approved for cancer treatment and marketed as Erbitux. Accordingly, the single-chain variable fragment (scFv) derived from cetuximab was used as the extracellular antigen-recognizing domain in a second-generation murine chimeric antigen receptor (CAR). In addition to cetuximab-derived scFv, the CAR included a murine CD28 domain—comprising the hinge, transmembrane and intracellular regions—followed by the intracellular signaling domain of murine CD3ζ. A retroviral vector was engineered to co-express the CAR and green fluorescent protein (GFP) from a single transcript using an internal ribosome entry site (IRES), with GFP serving as a fluorescent reporter for transduction efficiency (Figure 1B).

### 2.2. Engineering of EGFR mCAR T Cells

**Figure 2:**
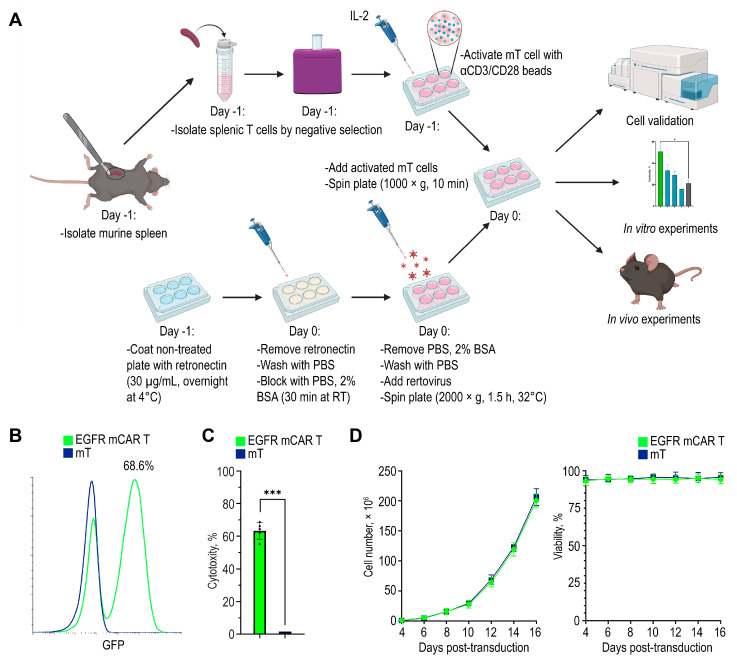
Generation of murine EGFR CAR T cells. (**A**) Workflow for EGFR mCAR T cell production, including T cell isolation, activation, retroviral transduction, and subsequent in vitro and in vivo experiments created with BioRender (https://www.biorender.com/ (accessed on 1 September 2025)). (**B**) Representative flow cytometry histogram showing the transduction efficiency of murine T cells with the EGFR-targeted murine CAR construct. (**C**) Summary of independent transduction preparations: percentage of GFP^+^ cells in each preparation. (**D**) Expansion (**left**) and viability (**right**) of EGFR mCAR T cells compared to non-transduced T cells over 14 days of culture. * *p* < 0.05 and *** *p* < 0.001.

Total splenic murine T cells were purified, activated with aCD3/CD28 beads, seeded on retronectin-coated plates, and transduced with retroviral particles (Figure 2A). Transduction efficiency was assessed by flow cytometry, yielding approximately 70% GFP-positive murine CAR T cells targeting the human/murine hybrid EGFR (EGFR mCAR T cells) (Figure 2B). The outcomes of independent transductions are summarized as the percentage of GFP^+^ cells per replicate (Figure 2C). Cell expansion was monitored over 14 days by counting viable cells every 48 h (Figure 2D left). Viability, assessed by trypan blue exclusion using an automated cell counter, remained above 90% throughout culture (Figure 2D right).

### 2.3. EGFR mCAR T Cells Exhibit Antigen-Specific Cytotoxicity and Pro-Inflammatory Cytokines Secretion In Vitro

**Figure 3:**
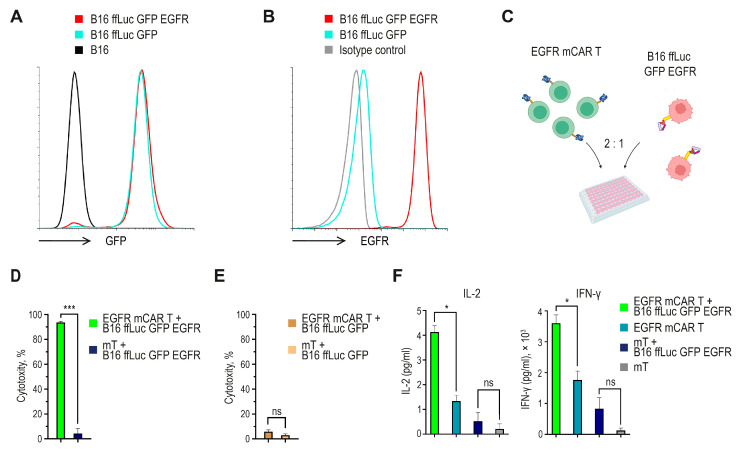
In vitro functional characterization of EGFR mCAR T cells. (**A**) Flow cytometric analysis of B16 cells transduced with a lentiviral vector encoding ffLuc and GFP. (**B**) Flow cytometric analysis of B16 ffLuc GFP cells further transduced with a lentiviral vector encoding the human/murine hybrid EGFR. (**C**) Experimental design of the cytotoxicity assay created with BioRender (https://www.biorender.com/ (accessed on 1 September 2025)). (**D**) Antigen-specific cytotoxicity of EGFR mCAR T cells against B16 ffLuc GFP EGFR target cells after 24 h of co-culture, calculated as the percentage decrease in bioluminescence relative to target-only control wells. (**E**) Lack of antigen-independent cytotoxicity. EGFR mCAR T or mT cells co-cultured with B16 ffLuc GFP (EGFR-negative) targets; cytotoxicity calculated as the percent decrease in bioluminescence relative to target-only wells. (**F**) Secretion of IL-2 and IFN-γ was detected in culture supernatants after 24 h of co-culture with or without antigen-positive tumor cells. ns (not significant, *p* ≥ 0.05), * *p* < 0.05 and *** *p* < 0.001.

To establish a target system for functional assays, B16 melanoma cells were first transduced with a lentiviral vector encoding firefly luciferase and GFP, followed by transduction with a lentiviral vector encoding a human/murine hybrid EGFR (next as EGFR). Flow cytometry confirmed robust GFP expression in reporter-positive cells and stable surface expression of EGFR, whereas parental B16 cells served as a negative control (Figure 3A,B). Detectable EGFR expression was further confirmed by Western blot analysis (Figure S1).

To evaluate the effector function of EGFR mCAR T cells, they were co-cultured with the engineered target melanoma cells (Figure 3C). Tumor cell viability was quantified by measuring luminescence from live target cells, and culture supernatants were collected for cytokine analysis. EGFR mCAR T cells demonstrated robust, antigen-dependent cytotoxic activity, resulting in specific lysis of more than 90% of EGFR-expressing tumor cells after 24 h of co-incubation (Figure 3D). As a negative control, EGFR mCAR T and mT cells were co-cultured with B16 ffLuc GFP target cells lacking EGFR expression, which did not result in a significant reduction in bioluminescence, indicating minimal antigen-independent cytotoxicity (Figure 3E). Consistently, cytokine secretion was observed only in response to antigen-positive targets. Co-culture with B16 ffLuc GFP EGFR cells for 24 h induced the secretion of approximately 4 pg/mL of IL-2 and 3.5 × 10^3^ pg/mL of interferon-gamma (IFN-γ) by EGFR mCAR T cells, while no significant cytokine release was detected in cultures without antigen-positive tumor cells (Figure 3F). Together, these data confirm that the engineered EGFR mCAR T cells are capable of mediating antigen-specific cytotoxicity and inducing pro-inflammatory cytokine production in vitro.

### 2.4. Optimizing Tumor Cell Dose and Early Assessment of Combined EGFR mCAR T Cell Therapy with Chemotherapy

**Figure 4:**
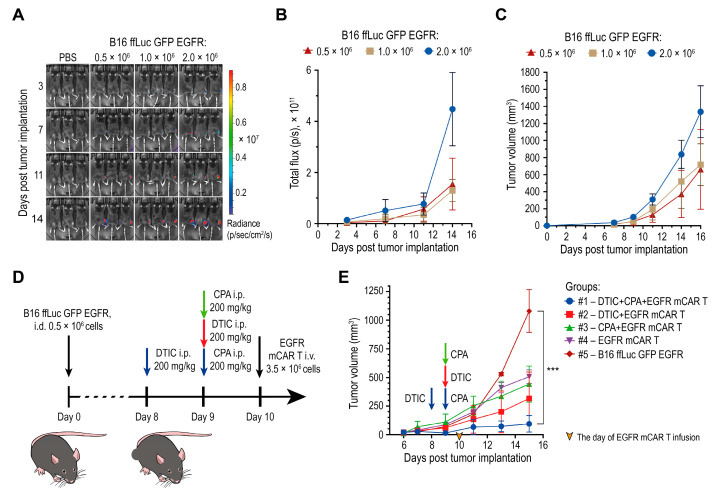
Chemotherapy with DTIC combined with EGFR mCAR T cell therapy after pre-conditioning with CPA delays tumor growth. (**A**) Bioluminescence imaging of engraftment and progression of B16 ffLuc GFP EGFR tumors following i.d. implantation of varying cell doses. (**B**,**C**) Quantification of the ROI bioluminescent signal (**B**) and tumor volume (**C**) for each implantation dose. (**D**) Experimental timeline for the preliminary in vivo assessment of mCAR T therapy with or without preceding chemotherapy. (**E**) Tumor growth kinetics of B16 ffLuc GFP EGFR tumors over 16 days under different combinatorial treatment regimens with EGFR mCAR T cells and chemotherapeutic agents. *** *p* < 0.001.

To determine optimal conditions for subsequent therapeutic studies, B16 ffLuc GFP EGFR tumor cells were implanted intradermally (i.d.) into female C57BL/6 mice at doses of 0.5 × 10^6^, 1 × 10^6^, or 2 × 10^6^ cells per mouse (*n* = 3 per group). Tumor progression was monitored by bioluminescence imaging following intraperitoneal (i.p.) injection of D-luciferin (3 mg per mouse) and by caliper measurements (Figure 4A–C). Progressive tumor growth was observed beginning on day 9, and mean tumor volumes on day 11 reached 118, 195, and 312 mm^3^ for the 0.5 × 10^6^, 1 × 10^6^, and 2 × 10^6^ cell doses, respectively (Figure 4C). Based on these growth kinetics, an inoculation dose of 0.5 × 10^6^ cells and day 10 post-implantation were selected as the conditions for EGFR mCAR T cell infusion.

For evaluation of the combined therapeutic regimen, including DTIC, EGFR mCAR T cells, and CPA, mice (*n* = 15) were inoculated i.d. with 0.5 × 10^6^ B16 ffLuc GFP EGFR cells and randomized into five groups (*n* = 3 per group): #1 DTIC + CPA + EGFR mCAR T; #2 DTIC + EGFR mCAR T; #3 CPA + EGFR mCAR T; #4 EGFR mCAR T alone; and #5 untreated control (Figure 4D). In group #1, DTIC (200 mg/kg, i.p.) was administered on day 8 and CPA (200 mg/kg, i.p.) on day 9. In group #2, the same dose of DTIC was administered on day 9. In group #3, the same dose of CPA was administered on day 9. In groups #1–#4, EGFR mCAR T cells (3.5 × 10^6^, i.v.) were infused on day 10.

Tumor growth was subsequently monitored by caliper measurements. From day 13, group #1 exhibited the most pronounced inhibition of tumor growth, while group #2 also demonstrated a significant delay in tumor progression. Group #3 showed only a modest improvement compared to group #4, indicating a supportive but not synergistic role of CPA in promoting CAR T cell activity. Tumors in untreated mice grew exponentially. Overall, the combined use of DTIC and CPA prior to EGFR mCAR T cell infusion produced maximal antitumor efficacy, suggesting a synergistic effect of dual-drug conditioning in enhancing EGFR mCAR T cell function (Figure 4E). These results were further validated in subsequent experiments that included additional control groups.

### 2.5. Combination of Dacarbazine and EGFR mCAR T Cell Therapy After Pre-Conditioning with Cyclophosphamide Leads to Robust Tumor Control and Extended Survival of Animals In Vivo

**Figure 5:**
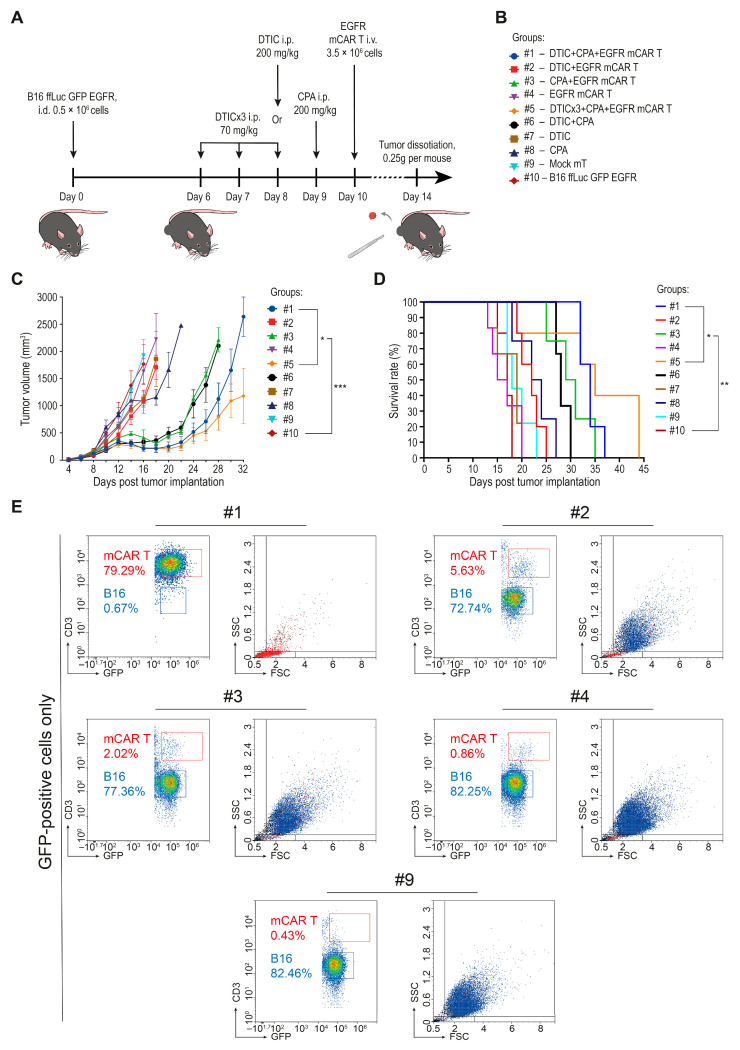
Combination of dacarbazine and EGFR mCAR T cell therapy after pre-conditioning with cyclophosphamide. (**A**) Schematic representation of the extended in vivo protocol outlining chemotherapeutic conditioning regimens and the timing of EGFR mCAR T cell administration across all groups. (**B**) Summary of experimental groups and treatment allocations. (**C**) Tumor growth kinetics of B16 ffLuc GFP EGFR tumors over 32 days across all treatment groups. (**D**) Kaplan–Meier survival analysis across all treatment groups over 44 days. (**E**) Quantification of B16 ffLuc GFP EGFR tumor cells and EGFR mCAR T cells based on expression of GFP and morphological characteristics following tumor tissue dissociation. * *p* < 0.05, ** *p* < 0.01, and *** *p* < 0.001; #1—group #1 (DTIC + CPA + mCAR T); #2—group #2 (DTIC + mCAR T); #3—group #3 (CPA + mCAR T); #4—group #4 (mCAR T only); #9—group #9 (mock mT cells).

A total of 0.5 × 10^6^ B16 ffLuc GFP EGFR tumor cells were injected i.d. into the right flank of 56 female C57BL/6 mice on day 1 (Figure 5A). Mice were then randomized into the following treatment groups: #1: DTIC + CPA + mCAR T (*n* = 6); #2: DTIC + mCAR T (*n* = 6); #3: CPA + mCAR T (*n* = 5); #4: mCAR T only (*n* = 6); #5: DTICx3 + CPA + mCAR T (*n* = 6); #6: DTIC + CPA (*n* = 5); #7: DTIC only (*n* = 6); #8: CPA only (*n* = 5); #9: Mock mT cells (*n* = 6); #10: Untreated control (*n* = 5) (Figure 5B).

Chemotherapy was administered i.p. at 200 mg/kg in all groups, except for group #5, in which DTIC was given in a fractionated, used in clinical practice, regimen at a dose of 70 mg/kg on days 6, 7, and 8 [27,28,29,30]. EGFR mCAR T cells were administered i.v. via the tail vein at a dose of 3.5 × 10^6^ GFP-positive cells (5 × 10^6^ total T cells, including both transduced and non-transduced cells, per mouse).

Tumor volume curves began to diverge as early as 4 days post-infusion (Figure 5C). The most pronounced tumor suppression was observed in group #5. Intermediate antitumor effects were seen in groups #2, #3, and #6. Monotherapies with DTIC, CPA, or mCAR T cells alone (#4, #7 and #8) had limited impact on tumor progression. Notably, group #1 (single-dose DTIC) initially showed tumor suppression comparable to group #5 (fractionated DTIC), but by day 26, tumor growth had resumed in the former group, suggesting transient control. Consistent with preliminary data (Figure 4E), CPA pre-conditioning enhanced therapeutic efficacy, likely due to its lymphodepleting and immunomodulatory effects, which support early expansion and function of infused EGFR mCAR T cells.

To evaluate the impact of treatment on survival, Kaplan–Meier survival curves were generated (Figure 5D). The highest survival rate was observed in group #5, which had the largest number of surviving mice by day 36. Group #1 showed a similar survival trend, albeit with earlier tumor relapse. Intermediate survival outcomes were seen in groups #3 and #6. In contrast, group #2 showed survival rates comparable to those of the monotherapy groups (#4, #7, #8) and the control groups (#9, #10).

For qualitative assessment of tumor cell content, tumor specimens were collected on day 14 post-implantation from one representative mouse per group. After visual inspection and euthanasia, approximately 0.25 g of tumor tissue was excised from each selected mouse. Tumors were processed using standardized protocols for mechanical and enzymatic dissociation into single-cell suspensions, which were then analyzed by flow cytometry. A substantial reduction in tumor cell abundance (below 0.5% among cells in the tumor sample) was observed only when dacarbazine and cyclophosphamide were combined with mCAR T therapy, which also resulted in an increased presence of mCAR T cells within the tumor tissue. Other treatment regimens did not produce comparable changes and overall tumor cell proportions remained elevated, with the exception of a decrease in group #6 (DTIC + CPA), which may reflect nonspecific immune activation against the modified tumor cells following limited lymphodepletion (Figure 5E and Figure S2; Table S1).

To assess systemic toxicity and tumor-related burden, body weight was monitored across all groups (Figure S3). Weight changes were used as an integrative measure of physiological status, reflecting both therapeutic side effects and tumor burden. During the first 10–12 days, body weight remained stable across most groups, except for moderate reductions in chemotherapy-treated mice. The most pronounced weight loss occurred in group #5, likely due to the cumulative effects of fractionated DTIC, CPA, and mCAR T cell therapy. Moderate weight loss (1–1.5 g) was also observed in groups #2, #3, and #6 with no signs of clinical distress, indicating acceptable tolerability. In contrast, groups #9 and #10 exhibited weight gain, likely due to unlimited tumor progression contributing to total body mass.

Thus, weight loss in therapeutic groups appeared transient and indicative of active immune engagement, while weight gain in control groups correlated with unrestrained tumor growth.

Collectively, these findings support the conclusion that effective tumor control requires synergistic interaction between immunogenic tumor modulation by DTIC and ACT with EGFR mCAR T cells, following CPA-mediated lymphodepletion. Nevertheless, partial lethality observed even in the most effective groups highlights the need for further optimization of the chemotherapy regimen and consideration of repeated EGFR mCAR T cell dosing to achieve durable remission.

## 3. Discussion

CAR T cell therapy has demonstrated remarkable clinical efficacy in B cell leukemias and lymphomas, resulting in durable remissions in a substantial proportion of patients [31,32]. However, its application to solid tumors, including melanoma, presents several unique challenges. These include substantial intratumoral heterogeneity, a scarcity of unique TAA, the presence of an immunosuppressive TME, limited trafficking and persistence of effector CAR T cells within the TME, the necessity for integration with conventional therapies, and the management of systemic toxicity [33,34].

In addition to CAR T cells, several therapeutic strategies are well established in melanoma and offer potential for synergistic combinations. Immune checkpoint inhibitors (ICIs), such as pembrolizumab, have significantly improved survival in advanced melanoma. The ability of ICIs to relieve inhibitory signaling may also enhance CAR T cell persistence and function [35]. Targeted therapies against BRAF V600 mutations, including combined BRAF/MEK inhibition, yield rapid responses and can remodel the TME in ways that might facilitate CAR T cell infiltration [36]. Most recently, tumor-infiltrating lymphocyte therapy with Lifileucel received Food and Drug Administration accelerated approval, becoming the first cellular immunotherapy approved for melanoma. This underscores both the promise and the complexity of adoptive cell therapies in solid tumors [37]. Collectively, these advances highlight the evolving landscape of melanoma treatment and the rationale for incorporating CAR T cells into multimodal regimens.

In the present study, we designed a strategy to overcome barriers in solid tumor therapy by employing a combinatorial regimen consisting of DTIC followed by infusion of engineered EGFR mCAR T cells after lymphodepleting pre-conditioning with CPA, using a B16 melanoma model.

Previous studies have explored combining CAR T cell therapy with chemotherapy in melanoma and other solid tumors. Lymphodepleting regimens using CPA and fludarabine are widely used to enhance CAR T cell engraftment and persistence [22]. In parallel, multiple antigen-specific CAR T cells have been developed and tested in preclinical melanoma models. For instance, TYRP1-directed CAR T cells effectively controlled tumor growth in xenograft models [38], and CSPG4-targeted CAR T cells demonstrated potent cytotoxicity and feasibility for clinical-scale mRNA-based manufacturing [39]. While these studies confirmed the antitumor potential of CAR T cells in melanoma, they were conducted as monotherapies. Thus, the therapeutic benefit of combining CAR T cell therapy with cytotoxic chemotherapy remains largely unexplored.

Alkylating agents such as temozolomide and DTIC have primarily been evaluated as tumor sensitizers, capable of inducing ICD and enhancing presentation of TAA [23,40], yet their integration with CAR T cell therapy has not been studied.

Our study demonstrates for the first time that sequential administration of DTIC, followed by EGFR mCAR T cell infusion after CPA pre-conditioning, results in significant antitumor efficacy in a syngeneic melanoma model. This combined regimen was associated with improved tumor control and prolonged survival. Specifically, the combination resulted in an approximately 80% reduction in average tumor volume by day 16 and roughly doubling of median survival. Repeated DTIC administration was most effective, inducing sustained tumor regression up to day 20 post-treatment. Importantly, none of the monotherapies (DTIC, CPA, or EGFR mCAR T cells) produced significant antitumor effects, highlighting the synergistic potential of the three-component regimen.

Our findings align with growing evidence that supports chemotherapeutic modulation of the TME as a means to enhance the efficacy of ACT. In addition to its direct cytotoxic effects, DTIC induces a tumor stress response that upregulates NKG2D ligands and promotes IFN-γ secretion, thereby enhancing immune recognition and effector lymphocyte recruitment [16]. CPA reduces Treg and MDSC populations, alleviating immunosuppressive pressure and fostering an environment more permissive to CAR T cell expansion and function [41]. Together, DTIC and CPA likely synergize by increasing TAA availability and immune visibility while simultaneously removing immunoregulatory barriers, thereby creating a more favorable microenvironment for CAR T cell infiltration, survival, and antitumor activity. This coordinated sequence of effects likely underpins the durable therapeutic response observed in our model.

Despite the observed therapeutic benefit, several limitations should be noted. Conceptually, neither chemotherapy nor CAR T cell therapy represents a standard treatment for melanoma. Curative surgery remains the mainstay for early-stage disease, while adjuvant or neoadjuvant immune checkpoint blockade is the current standard for advanced melanoma. Furthermore, EGFR is generally not considered a relevant therapeutic target in this tumor type. Nevertheless, EGFR expression has been documented in a subset of melanoma cells and is associated with more aggressive, therapy-resistant phenotypes [24,42,43,44,45]. In the present study, melanoma was selected primarily as a mechanistic model to investigate antigen-dependent cytotoxicity and immune interactions in a well-characterized tumor system. While the translational relevance of EGFR-directed therapy may be greater in tumors such as non-small cell lung cancer, colorectal cancer, glioblastoma, or breast cancer, the findings from our model provide insights into EGFR-targeted immune strategies that may be extended to EGFR-expressing cancers. An additional important limitation of our study is the use of viral vectors for the genetic modification of both tumor cells and CAR T cells. Although such transduction is a widely used and efficient method for stable gene delivery, it may increase the immunogenicity of the modified cells, especially in the context of chemotherapy-induced lymphodepletion. This could potentially affect CAR T cell persistence and function, as well as contribute to immune-mediated clearance of engineered tumor cells, thereby influencing the interpretation of therapeutic efficacy. Although our data suggest that CPA-mediated lymphodepletion contributes to the enhanced efficacy of combination therapy, the precise immunological mechanisms underlying this effect remain to be fully clarified. In the present study, we did not perform a detailed flow-cytometric characterization of tumor-infiltrating immune subsets, which limits our ability to directly assess how CPA influences the tumor microenvironment or CAR T cell dynamics in vivo. Previous work in murine models has shown that CPA can transiently deplete endogenous lymphocytes, promote homeostatic cytokine release, and facilitate the engraftment and activation of adoptively transferred T cells, providing a plausible explanation for the trends observed in our study [22,46,47,48,49,50]. Flow cytometry confirmed the presence and persistence of CAR T cells within the tumor. However, functional analyses such as Ki-67 staining or cytokine measurements were not performed, limiting insight into in vivo CAR T cell activation. Future studies including these assessments will help clarify the mechanisms of CAR T cell-mediated tumor control. In particular, deeper profiling of tumor-infiltrating immune subsets, CAR T cell activation status, and potential mechanisms of antigen escape (including antigen modulation or loss) remains necessary to fully understand the immunological events driving treatment outcome. Moreover, as melanoma encompasses a heterogeneous spectrum of subtypes, including metastatic forms that may respond differently to immunotherapy and chemotherapy, it will be important for future research to validate these findings across diverse melanoma models. This will help determine the robustness and broader applicability of the observed therapeutic effects.

Nevertheless, the combined use of DTIC, CPA, and CAR T cells leverages components with established clinical relevance, suggesting that this approach may warrant further investigation in a translational context. In this setting, repeated DTIC administration could contribute to increased tumor immunogenicity, while a single CPA dose may provide sufficient lymphodepletion to support CAR T cell engraftment with acceptable toxicity. Clinically equivalent doses of DTIC and CPA are generally well tolerated [28,29,30]. Moreover, DTIC has been shown to upregulate PD-L1 expression on tumor cells, suggesting that the addition of PD-1/PD-L1 blockade could prevent CAR T cell exhaustion and further prolong remission duration [51].

In summary, this study demonstrates that treatment of melanoma-bearing mice with DTIC and CPA effectively transforms poorly immunogenic melanoma into a tumor susceptible to CAR T cell-mediated cytotoxicity. The combination of enhanced tumor immunogenicity, lymphodepletion, and improved immune accessibility creates a favorable microenvironment for CAR T cell infiltration, expansion, and sustained activity, resulting in robust tumor regression and extended survival without significant toxicity. These findings support the clinical evaluation of this regimen in treatment-refractory solid tumors and highlight opportunities to further improve outcomes by integrating additional immunomodulatory agents.

## 4. Materials and Methods

### 4.1. Cells and Culture Conditions

The HEK293T (ATCC, Manassas, VA, USA) and B16 (ATCC, Manassas, VA, USA) cell lines were cultured in Full DMEM (Advanced DMEM (Gibco, Waltham, MA, USA) supplemented with 10% fetal bovine serum (FBS) (Hyclone, Logan, UT, USA), 100 U/mL penicillin (Gibco, Waltham, MA, USA), 100 µg/mL streptomycin (Gibco, Waltham, MA, USA) and 2 mM GlutaMAX (Gibco, Waltham, MA, USA)) at 37 °C, 5% CO_2_ up to 80–90% confluency. Murine T and CAR T cells were cultured in Full RPMI (Advanced RPMI-1640 (Gibco, Waltham, MA, USA) supplemented with 10% FBS (Hyclone, Logan, UT, USA), 100 U/mL penicillin, 100 µg/mL streptomycin, 2 mM GlutaMAX, 0.1 µg/mL (IL-2) (Sci Store, Moscow, Russia), and 50 µM 2-mercaptoethanol (Sigma-Aldrich, St. Louis, MO, USA)) at 37 °C, 5% CO_2_, maintaining a cell density of 2 × 10^6^ cells/mL. Viable cell counts and viability assessments were performed every 48 h using a DeNovix CellDrop BF cell counter (DeNovix, Wilmington, DE, USA) with 0.04% trypan blue solution for exclusion assay. All lines were screened monthly for mycoplasma contamination using the MycoReport kit (Evrogen, Moscow, Russia).

### 4.2. Flow Cytometry and Cell Sorting

For immunophenotyping, 3 × 10^5^ cells were precipitated (300× *g*, 5 min, room temperature (RT)) and resuspended in 25 µL phosphate-buffered saline (PBS) (Paneco, Moscow, Russia) supplemented with 5% newborn bovine serum (NBS) (Globe Kang, Qinhuangdao, China). Cells were stained with 0.25 µL fluorochrome-conjugated antibodies (Appendix A, Table A1) for 30 min at 4 °C, washed once with 1 mL cold PBS (300× *g*, 5 min), and resuspended in 150 µL PBS. Samples were acquired on an ACEA NovoCyte 3000 cytometer (Agilent, Santa Clara, CA, USA) and analyzed using NovoExpress software version 1.6.1 (Agilent, Santa Clara, CA, USA) and the free online software Floreada.io (https://floreada.io/ (accessed on 10 September 2025)). For cell sorting, stained or GFP^+^ cells were processed on a SH800S cell sorter (Sony Biotechnology, Tokyo, Japan).

### 4.3. Chimeric Antigen Receptor Constructs

The EGFR mCAR comprised a single-chain variable fragment (scFv, derived from the monoclonal antibody cetuximab) targeting human/murine hybrid EGFR, fused to the murine CD28, including hinge, transmembrane and intracellular co-stimulatory parts, followed by the intracellular piece of murine CD3ζ. The synthetic CAR gene was cloned by PCR amplification with primers containing restriction sites, restriction digest, and ligation (restriction enzymes and ligase from New England Biolabs, Ipswich, MA, USA) into the retroviral vector pMSCV (a gift from Dr. Reya, Addgene, #20672) right before IRES followed by the reporter gene of GFP. A synthetic gene of human/murine hybrid EGFR (BioInnLabs, Rostov-on-Don, Russia) was inserted into the pLV3 lentiviral vector (Clontech, San Jose, CA, USA) with the same method. All plasmids were verified by Sanger sequencing (Evrogen, Moscow, Russia).

### 4.4. Lentiviruses and Retroviruses Production

HEK293T cells were seeded at 6.3 × 10^6^ cells in 100 mm dishes in Full DMEM. 2 h before transfection, the medium was replaced with Full DMEM without a/a. Transfections were performed using GenJect-39 (Molecta, Moscow, Russia) at a DNA/reagent ratio of 1 µg:4 µL. For each dish, 500 µL Opti-MEM (Gibco, Waltham, MA, USA) was used to prepare: (1) Plasmid mix (for lentiviral particles: pMDLg/pRRE, pRSV-Rev, pMD2.G (all are gifts from Dr. Trono, Addgene #12251, #12253, #12259), and transfer plasmid; for retroviral particles: pCL-ECO (a gift from Dr. I. Verma, Addgene #12371), and transfer plasmid. (2) GenJect-39 solution, calculated on total DNA weight. Each component was incubated separately for 10 min at RT, then combined and incubated for an additional 15 min before dropwise addition to the cells. After 6 h, the medium was replaced with 10 mL Full DMEM, and cells were incubated for 48 h at 37 °C, 5% CO_2_. Viral supernatants were harvested 48 h after medium replacement. The collected supernatant was centrifuged twice at 300× *g* and 4500× *g* for 5 min at 10 °C to remove large and tiny cell debris. The clarified supernatant was transferred into fresh tubes and mixed with Lenti-X Concentrator (Takara, Osaka, Japan) for downstream concentration, according manufacturer’s manual. After overnight incubation at 4 °C, samples were centrifuged at 1500× *g* for 45 min at 4 °C. Supernatants were discarded, and viral pellets were gently resuspended in 100 µL of Advanced RPMI-1640. Aliquots were snap-frozen in liquid nitrogen and stored at −80 °C until use.

### 4.5. Lentiviral Transduction of B16 Melanoma Cells

One day prior to infection, B16 cells were seeded at 0.5 × 10^6^ cells per well in 6-well plates. On the day of transduction, half of the medium was replaced with Full DMEM containing 10 µg/mL Polybrene (Millipore, Burlington, MA, USA). An equal volume of thawed lentiviral supernatant was added, and cells were incubated overnight at 37 °C, 5% CO_2_. Medium was then removed and replaced with fresh Full DMEM. Transduction efficiency was evaluated on days 3–4 by flow cytometry using Cetuximab conjugated in-house with allophycocyanin and EGFR^+^ or GFP^+^ cells were enriched by FACS when necessary.

### 4.6. Western Blotting

Cell pellets of 5 × 10^6^ B16, B16 ffLuc GFP and B16 ffLuc GFP EGFR cells were lysed with RIPA lysis buffer (50 mM Tris HCl, 150 mM NaCl, 1.0% (*v*/*v*) NP-40, 0.5% (*w*/*v*) so-dium deoxycholate, 1.0 mM EDTA, 0.1% (*w*/*v*) SDS, and 0.01% (*w*/*v*) sodium azide (pH 7.4)). Cell lysates were mixed with 2× sample buffer (125 mM Tris HCl (pH 6.8), 4% (*w*/*v*) SDS, 20% (*v*/*v*) glycerol, 100 mM DTT, 0.01% bromophenol blue) and heated at 90 °C for 10 min (all chemicals are from Sigma-Aldrich, St. Louis, MO, USA). Samples were separated by SDS-PAGE on an 8% polyacrylamide gel, transferred to a nitrocellulose membrane (Bio-Rad, Hercules, CA, USA), and incubated with an EGF Receptor Rabbit Monoclonal Antibody and HRP-conjugated Goat anti-Rabbit IgG (H + L) Secondary Antibody, according to the manufacturers’ protocols. A Direct-Blot HRP anti-β-actin Antibody was used as a positive loading control. The presence of EGFR and actin was assessed on a VersaDoc 4000 MP imaging system (Bio-Rad, Hercules, CA, USA) using an Enhanced Chemiluminescence kit (Bio-Rad, Hercules, CA, USA).

### 4.7. Isolation and Transduction of Murine T Cells

Spleens from female C57BL/6 mice (6–8 weeks old, 19–21 g; from the Unique Research Unit Bio-Model of the IBCh, RAS; the Bioresource Collection–Collection of SPF-Laboratory Rodents for Fundamental, Biomedical and Pharmacological Studies, no. 075-15-2025-486) were collected into cold PBS and mechanically dissociated using a syringe plunger. The suspension was filtered through a 70 µm cell strainer (SPL Lifesciences, Pocheon-si, Republic of Korea), pelleted (300× *g*, 10 min, 4 °C), and treated with 1 mL ammonium-chloride-potassium (ACK) (Gibco, Waltham, MA, USA) lysing buffer for 3 min at RT to lyse erythrocytes. Lysis was stopped by adding PBS and cells were pelleted during the second centrifugation (300× *g*, 10 min, 4 °C). Untouched T cells were isolated using the Dynabeads Untouched Mouse T Cell Isolation Kit (Invitrogen, Carlsbad, CA, USA) per the manufacturer’s instructions. Purified T cells were resuspended in Full RPMI, then activated with Dynabeads Mouse T-Activator aCD3/CD28 (Invitrogen, Carlsbad, CA, USA) at a 2:1 bead-to-cell ratio. Activated cells were cultured at 37 °C, 5% CO_2_. For retroviral transduction, non-treated 6-well plates were pre-coated with Retronectin (30 µg/mL; Takara, Osaka, Japan) in PBS for 2 h at RT. Fifty-fold concentrated retroviral supernatant (150 µL) was diluted in 2 mL Full RPMI, added to each well, and spinoculated at 2000× *g* for 2 h at 32 °C. Wells were then washed once with PBS, and then 2.5 × 10^6^ activated T cells were added and centrifuged at 1000× *g* for 10 min. Cultures were maintained in Full RPMI and medium was refreshed after 48 h. Transduction efficiency was assessed 48 h later by flow cytometry.

### 4.8. Expansion Assay of EGFR mCAR T Cells

EGFR mCAR T cells (2.5 × 10^5^ per well) were plated in 24-well plates in Full RPMI. Cultures were maintained at 37 °C, 5% CO_2_. When cell density reached 2 × 10^6^ cells/mL, cultures were split into larger vessels to maintain optimal growth conditions. Total cell counts and viability were determined every 48 h for 14 days using 0.4% trypan blue exclusion assay and measured on a DeNovix CellDrop BF cell counter (DeNovix, Wilmington, DE, USA).

### 4.9. Cytotoxicity Assay

EGFR mCAR T cells were co-cultured with B16 ffLuc GFP target cells expressing EGFR. Target cells were seeded at 1.5 × 10^4^ cells per well in white 96-well flat-bottom plates (SPL Lifesciences, Pocheon-si, Republic of Korea) 24 h prior to the addition of effector cells, and cultured in Full RPMI without IL-2 at 37 °C, 5% CO_2_. Effector and target cells were mixed at E:T ratios of 2:1, 1:1, 1:2, and 1:4, with unstimulated murine T cells as a negative control. After 24, 48, or 72 h of incubation, plates were centrifuged at 500× *g* for 5 min. The supernatants were removed, and 1 µL of D-luciferin (Molecta, Moscow, Russia) stock solution (15 mg/mL in PBS) was added to 100 µL of fresh PBS in each well. Plates were incubated in the dark at RT for 5 min, and luminescence was measured on a Varioskan Lux plate reader (Thermo Fisher Scientific, Waltham, MA, USA). Percent cytotoxicity was calculated as: C = (1 − (S_exp_/S_ctrl_)) × 100% where S_exp_ is the luminescence in effector-containing wells and S_ctrl_ is the luminescence in wells containing target cells only.

### 4.10. Enzyme-Linked Immunosorbent Assay (ELISA)

Cytokine secretion of IL-2 and IFN-γ was quantified following co-culture of mCAR T cells with target cells. Effector and target cells were incubated at an E:T ratio 2:1 in 96-well flat-bottom plates (Nest, Wuxi, China) for 24 h at 37 °C, 5% CO_2_. After incubation, cultures were centrifuged at 500× *g* for 5 min at 20 °C. Cell-free supernatants were either assayed immediately or stored at −20 °C for up to 14 days. Concentrations of IL-2 and IFN-γ were measured using mouse IFN-γ and mouse IL-2 ELISA kits (Elabscience, Wuhan, China) according to the manufacturer’s sandwich-ELISA protocol. Briefly, 50 µL of standards or samples were added to antibody-coated wells and incubated for 2 h at RT. Wells were washed three times with a wash buffer, followed by 1 h incubation with biotinylated detection antibody and subsequent 30 min incubation with horseradish peroxidase-conjugated streptavidin. After additional washes, 100 µL of TMB substrate (Thermo Fisher Scientific, Waltham, MA, USA) was added and incubated for 10–15 min at RT in the dark, and the reaction was stopped with 50 µL of stop solution. Optical density was read on a Varioskan Lux plate reader (Thermo Fisher Scientific, Waltham, MA, USA) at 450 nm. Cytokine concentrations were counted by standard samples.

### 4.11. Animal Studies

All procedures were approved by the Bioethics Commission of the Shemyakin–Ovchinnikov Institute of Bioorganic Chemistry, RAS. Female C57BL/6 mice (6–8 weeks old, 19–21 g; from the Unique Research Unit Bio-Model of the IBCh, RAS; the Bioresource Collection—Collection of SPF-Laboratory Rodents for Fundamental, Biomedical and Pharmacological Studies, no. 075-15-2025-486)) were housed under specific-pathogen free conditions, which included a controlled environment with appropriate temperature (21–24 °C), humidity (40–60%), and 12 h light/dark cycles. On day 0, 5 × 10^5^ B16 ffLuc GFP EGFR cells in 50 µL PBS were injected i.d. into the right flank. Tumor growth was monitored every other day by caliper measurement, and weekly by bioluminescence imaging (IVIS Lumina III, PerkinElmer, Shelton, CT, USA) following i.p. administration of D-luciferin (3 mg per mouse) under isoflurane anesthesia. Tumor dimensions were measured with digital calipers, and volume was calculated as: Volume = (length × width^2^)/2. Measurements were performed at regular intervals by trained personnel. While tumor photographs were not collected during this experimental series, the caliper-based measurements provide a reliable quantitative assessment of tumor progression and treatment effects. Body weight and clinical status were recorded.

From day 6 post-implantation, mice underwent chemotherapy with DTIC (Dacarbazine-Rus, Apothecon Pharmaceuticals Pvt. Ltd., Valodara, India) and lymphodepletion with CPA (Endoxan, Baxter, Unterschleißheim, Germany). DTIC was administered i.p. either as a single dose of 200 mg/kg or as a fractionated regimen of 70 mg/kg daily for three consecutive days, followed 24 h later by CPA (200 mg/kg, i.p.). On day 10, 3.5 × 10^6^ EGFR mCAR T cells in 50 µL PBS were administered i.v. Mice were euthanized when tumors reached ≥2000 mm^3^ (or the equivalent of ~10% body weight), in accordance with our ethical guidelines, or upon exhibiting signs of distress.

### 4.12. Tumor Dissociation

On day 14, 0.25 g of tumors from one mouse per group were excised aseptically, minced in a total of 2 mL of medium, prepared as a 1:1 mixture of Full DMEM and Full RPMI, and incubated for 40 min at 37 °C, 8% CO_2_ in 2 mL enzyme cocktail containing collagenase I + IV (1 mg each; 14.5 U) (both are from Paneco, Moscow, Russia), hyaluronidase (2.6 U) (Microgen, Moscow, Russia), and DNase I (0.25 mg) (Thermo Fisher Scientific, Waltham, MA, USA). Tissue was gently triturated, filtered sequentially through 100, 70, and 40 µm nylon cell strainers (all are from SPL Lifesciences, Pocheon-si, Republic of Korea), and washed in PBS (300× *g*, 15 min, RT). Erythrocytes were lysed with ACK buffer for 1 min at RT, followed by a wash (300× *g*, 7 min, RT). The resulting cell pellet was resuspended in 1 mL PBS, counted, and immediately used for immunostaining and flow cytometry analysis.

### 4.13. Statistical Analysis

All statistical analyses were performed using GraphPad Prism software version 10.2.3 (GraphPad Software, San Diego, CA, USA). Data are presented as mean ± standard deviation (SD). Unless otherwise specified, all experiments were conducted in a minimum of three independent replicates. Comparisons between two groups were made using unpaired two-tailed Student’s *t*-tests, while multi-group or repeated-measures data were analyzed by two-way ANOVA with appropriate post hoc tests. Survival curves were generated by the Kaplan–Meier method and compared using the log-rank test. A *p*-value of <0.05 was considered statistically significant. Significance levels are denoted as: ns (not significant, *p* ≥ 0.05), * *p* < 0.05, ** *p* < 0.01, *** *p* < 0.001, and **** *p* < 0.0001.

## Data Availability

The original contributions presented in this study are included in the article and Supplementary Material. Further inquiries can be directed to the corresponding authors.

## Supplementary Materials

The following supporting information can be downloaded at: https://www.mdpi.com/article/10.3390/ijms27010189/s1.

## Abbreviations

The following abbreviations are used in this manuscript:

ACT	Adoptive cell therapy
CAR	Chimeric antigen receptor
CAR T cells	T cell modified with chimeric antigen receptor
CPA	Cyclophosphamide
DTIC	Dacarbazine
EGFR	Epidermal growth factor receptor
EGFR mCAR T cells	EGFR-specific murine CAR T cells
ELISA	Enzyme-linked immunosorbent assay
GFP	Green fluorescent protein
ICI	Immune checkpoint inhibitor
i.d.	Intradermal infusion
IFN-γ	Interferon-gamma
IL	Interleukin
i.p.	Intraperitoneal infusion
IRES	Internal ribosome entry site
i.v.	Intravenous infusion
MDSC	Myeloid derived suppressor cell
scFv	Single-chain variable fragment
TAA	Tumor-associated antigens
TIL	Tumor-infiltrating lymphocyte
TME	Tumor microenvironment
Treg	Regulatory T cell

## Appendix A

**Table A1:** Antibodies.

Antibody	Source	Identifier
Cetuximab-APC	in-house	Not Applicable
Anti-mouse CD3-APC	BioLegend (San Diego, CA, USA)	100235; RRID: AB_2561455
EGF Receptor Rabbit Monoclonal Antibody	Cell Signaling Technology (Danvers, MA, USA)	4267S; RRID: AB_2895042
Goat anti-Rabbit IgG (H + L) Secondary Antibody, HRP	Thermo Fisher Scientific (Waltham, MA, USA)	31460; RRID: AB_228341
Direct-Blot HRP anti-β-actin Antibody	BioLegend (San Diego, CA, USA)	664804; RRID: AB_2728496

## References

[1] Koury J., Lucero M., Cato C., Chang L., Geiger J., Henry D., Hernandez J., Hung F., Kaur P., Teskey G. (2018). Immunotherapies: Exploiting the Immune System for Cancer Treatment. J. Immunol. Res..

[2] D’Errico G., Machado H.L., Sainz B. (2017). A current perspective on cancer immune therapy: Step-by-step approach to constructing the magic bullet. Clin. Transl. Med..

[3] Newick K., O’Brien S., Moon E., Albelda S.M. (2017). CAR T Cell Therapy for Solid Tumors. Annu. Rev. Med..

[4] Mlecnik B., Bindea G., Angell H.K., Sasso M.S., Obenauf A.C., Fredriksen T., Lafontaine L., Bilocq A.M., Kirilovsky A., Tosolini M. (2014). Functional network pipeline reveals genetic determinants associated with in situ lymphocyte proliferation and survival of cancer patients. Sci. Transl. Med..

[5] Louis C.U., Savoldo B., Dotti G., Pule M., Yvon E., Myers G.D., Rossig C., Russell H.V., Diouf O., Liu E. (2011). Antitumor activity and long-term fate of chimeric antigen receptor-positive T cells in patients with neuroblastoma. Blood.

[6] Brown C.E., Alizadeh D., Starr R., Weng L., Wagner J.R., Naranjo A., Ostberg J.R., Blanchard M.S., Kilpatrick J., Simpson J. (2016). Regression of Glioblastoma after Chimeric Antigen Receptor T-Cell Therapy. N. Engl. J. Med..

[7] Salmon H., Franciszkiewicz K., Damotte D., Dieu-Nosjean M.C., Validire P., Trautmann A., Mami-Chouaib F., Donnadieu E. (2012). Matrix architecture defines the preferential localization and migration of T cells into the stroma of human lung tumors. J. Clin. Investig..

[8] Maggi E., Munari E., Landolina N., Mariotti F.R., Azzarone B., Moretta L. (2024). T cell landscape in the microenvironment of human solid tumors. Immunol. Lett..

[9] Pauken K.E., Sammons M.A., Odorizzi P.M., Manne S., Godec J., Khan O., Drake A.M., Chen Z., Sen D.R., Kurachi M. (2016). Epigenetic stability of exhausted T cells limits durability of reinvigoration by PD-1 blockade. Science.

[10] Chow A., Perica K., Klebanoff C.A., Wolchok J.D. (2022). Clinical implications of T cell exhaustion for cancer immunotherapy. Nat. Rev. Clin. Oncol..

[11] Batlle E., Massagué J. (2019). Transforming Growth Factor-β Signaling in Immunity and Cancer. Immunity.

[12] Kroemer G., Galluzzi L., Kepp O., Zitvogel L. (2013). Immunogenic cell death in cancer therapy. Annu. Rev. Immunol..

[13] Li N., Li Y., Li J., Tang S., Gao H., Li Y. (2025). Correlation of the abundance of MDSCs, Tregs, PD-1, and PD-L1 with the efficacy of chemotherapy and prognosis in gastric cancer. Lab. Med..

[14] Bracci L., Schiavoni G., Sistigu A., Belardelli F. (2014). Immune-based mechanisms of cytotoxic chemotherapy: Implications for the design of novel and rationale-based combined treatments against cancer. Cell Death Differ..

[15] Garrido F., Cabrera T., Aptsiauri N. (2010). “Hard” and “soft” lesions underlying the HLA class I alterations in cancer cells: Implications for immunotherapy. Int. J. Cancer.

[16] Hervieu A., Rébé C., Végran F., Chalmin F., Bruchard M., Vabres P., Apetoh L., Ghiringhelli F., Mignot G. (2013). Dacarbazine-mediated upregulation of NKG2D ligands on tumor cells activates NK and CD8 T cells and restrains melanoma growth. J. Investig. Dermatol..

[17] Lutsiak M.E., Semnani R.T., De Pascalis R., Kashmiri S.V., Schlom J., Sabzevari H. (2005). Inhibition of CD4(+)25+ T regulatory cell function implicated in enhanced immune response by low-dose cyclophosphamide. Blood.

[18] Kochenderfer J.N., Somerville R.P.T., Lu T., Shi V., Bot A., Rossi J., Xue A., Goff S.L., Yang J.C., Sherry R.M. (2017). Lymphoma Remissions Caused by Anti-CD19 Chimeric Antigen Receptor T Cells Are Associated with High Serum Interleukin-15 Levels. J. Clin. Oncol. Off. J. Am. Soc. Clin. Oncol..

[19] Philippova J., Shevchenko J., Alsalloum A., Fisher M., Alrhmoun S., Perik-Zavodskii R., Perik-Zavodskaia O., Lopatnikova J., Kurilin V., Volynets M. (2025). GD2-Specific CAR T Cells Demonstrate Potent and Targeted Anti-Tumor Efficacy Against Melanoma In Vitro and In Vivo. Front. Biosci..

[20] Giraudo L., Cattaneo G., Gammaitoni L., Iaia I., Donini C., Massa A., Centomo M.L., Basiricò M., Vigna E., Pisacane A. (2023). CSPG4 CAR-redirected Cytokine Induced Killer lymphocytes (CIK) as effective cellular immunotherapy for HLA class I defective melanoma. J. Exp. Clin. Cancer Res. CR.

[21] Zhang Z., Jiang C., Liu Z., Yang M., Tang X., Wang Y., Zheng M., Huang J., Zhong K., Zhao S. (2020). B7-H3-Targeted CAR-T Cells Exhibit Potent Antitumor Effects on Hematologic and Solid Tumors. Mol. Ther. Oncolytics.

[22] Wang A.X., Ong X.J., D’Souza C., Neeson P.J., Zhu J.J. (2023). Combining chemotherapy with CAR-T cell therapy in treating solid tumors. Front. Immunol..

[23] Karachi A., Yang C., Dastmalchi F., Sayour E.J., Huang J., Azari H., Long Y., Flores C., Mitchell D.A., Rahman M. (2019). Modulation of temozolomide dose differentially affects T-cell response to immune checkpoint inhibition. Neuro-Oncology.

[24] Sun Y., Yu H., Zhou Y., Bao J., Qian X. (2025). EGFR influences the resistance to targeted therapy in BRAF (V600E) melanomas by regulating the ferroptosis process. Arch. Dermatol. Res..

[25] Zhuang X., Wang Z., Fan J., Bai X., Xu Y., Chou J.J., Hou T., Chen S., Pan L. (2022). Structure-guided and phage-assisted evolution of a therapeutic anti-EGFR antibody to reverse acquired resistance. Nat. Commun..

[26] Shen J., Zou Z., Guo J., Cai Y., Xue D., Liang Y., Wang W., Peng H., Fu Y.X. (2022). An engineered concealed IL-15-R elicits tumor-specific CD8+T cell responses through PD-1-cis delivery. J. Exp. Med..

[27] Nair A.B., Jacob S. (2016). A simple practice guide for dose conversion between animals and human. J. Basic Clin. Pharm..

[28] Hervieu A., Mignot G., Ghiringhelli F. (2013). Dacarbazine mediate antimelanoma effects via NK cells. Oncoimmunology.

[29] Cohen A.D., Garfall A.L., Stadtmauer E.A., Melenhorst J.J., Lacey S.F., Lancaster E., Vogl D.T., Weiss B.M., Dengel K., Nelson A. (2019). B cell maturation antigen-specific CAR T cells are clinically active in multiple myeloma. J. Clin. Investig..

[30] Weide B., Eigentler T., Catania C., Ascierto P.A., Cascinu S., Becker J.C., Hauschild A., Romanini A., Danielli R., Dummer R. (2019). A phase II study of the L19IL2 immunocytokine in combination with dacarbazine in advanced metastatic melanoma patients. Cancer Immunol. Immunother. CII.

[31] Kearl T.J., Furqan F., Shah N.N. (2024). CAR T-cell therapy for B-cell lymphomas: Outcomes and resistance mechanisms. Cancer Metastasis Rev..

[32] Schuster S.J., Svoboda J., Chong E.A., Nasta S.D., Mato A.R., Anak Ö., Brogdon J.L., Pruteanu-Malinici I., Bhoj V., Landsburg D. (2017). Chimeric Antigen Receptor T Cells in Refractory B-Cell Lymphomas. N. Engl. J. Med..

[33] Yan T., Zhu L., Chen J. (2023). Current advances and challenges in CAR T-Cell therapy for solid tumors: Tumor-associated antigens and the tumor microenvironment. Exp. Hematol. Oncol..

[34] Kong Y., Li J., Zhao X., Wu Y., Chen L. (2024). CAR-T cell therapy: Developments, challenges and expanded applications from cancer to autoimmunity. Front. Immunol..

[35] Long G.V., Carlino M.S., McNeil C., Ribas A., Gaudy-Marqueste C., Schachter J., Nyakas M., Kee D., Petrella T.M., Blaustein A. (2024). Pembrolizumab versus ipilimumab for advanced melanoma: 10-year follow-up of the phase III KEYNOTE-006 study. Ann. Oncol. Off. J. Eur. Soc. Med. Oncol..

[36] Ascierto P.A., Dréno B., Larkin J., Ribas A., Liszkay G., Maio M., Mandalà M., Demidov L., Stroyakovskiy D., Thomas L. (2021). 5-Year Outcomes with Cobimetinib plus Vemurafenib in BRAFV600 Mutation-Positive Advanced Melanoma: Extended Follow-up of the coBRIM Study. Clin. Cancer Res. Off. J. Am. Assoc. Cancer Res..

[37] Tsai K.K., Komanduri K.V. (2025). Tumor-Infiltrating Lymphocyte Therapy for the Treatment of Metastatic Melanoma. Am. J. Clin. Dermatol..

[38] Hackett C.S., Hirschhorn D., Tang M.S., Purdon T.J., Marouf Y., Piersigilli A., Agaram N.P., Liu C., Schad S.E., de Stanchina E. (2024). TYRP1 directed CAR T cells control tumor progression in preclinical melanoma models. Mol. Ther. Oncol..

[39] Wiesinger M., März J., Kummer M., Schuler G., Dörrie J., Schuler-Thurner B., Schaft N. (2019). Clinical-Scale Production of CAR-T Cells for the Treatment of Melanoma Patients by mRNA Transfection of a CSPG4-Specific CAR under Full GMP Compliance. Cancers.

[40] Chen X., Habib S., Alexandru M., Chauhan J., Evan T., Troka J.M., Rahimi A., Esapa B., Tull T.J., Ng W.Z. (2024). Chondroitin Sulfate Proteoglycan 4 (CSPG4) as an Emerging Target for Immunotherapy to Treat Melanoma. Cancers.

[41] Madondo M.T., Quinn M., Plebanski M. (2016). Low dose cyclophosphamide: Mechanisms of T cell modulation. Cancer Treat. Rev..

[42] Boone B., Jacobs K., Ferdinande L., Taildeman J., Lambert J., Peeters M., Bracke M., Pauwels P., Brochez L. (2011). EGFR in melanoma: Clinical significance and potential therapeutic target. J. Cutan. Pathol..

[43] de Wit P.E., Moretti S., Koenders P.G., Weterman M.A., van Muijen G.N., Gianotti B., Ruiter D.J. (1992). Increasing epidermal growth factor receptor expression in human melanocytic tumor progression. J. Investig. Dermatol..

[44] Kreß J.K.C., Jessen C., Marquardt A., Hufnagel A., Meierjohann S. (2021). NRF2 Enables EGFR Signaling in Melanoma Cells. Int. J. Mol. Sci..

[45] Lee K.H., Suh H.Y., Lee M.W., Lee W.J., Chang S.E. (2021). Prognostic Significance of Epidermal Growth Factor Receptor Expression in Distant Metastatic Melanoma from Primary Cutaneous Melanoma. Ann. Dermatol..

[46] Bracci L., Moschella F., Sestili P., La Sorsa V., Valentini M., Canini I., Baccarini S., Maccari S., Ramoni C., Belardelli F. (2007). Cyclophosphamide enhances the antitumor efficacy of adoptively transferred immune cells through the induction of cytokine expression, B-cell and T-cell homeostatic proliferation, and specific tumor infiltration. Clin. Cancer Res. Off. J. Am. Assoc. Cancer Res..

[47] Gattinoni L., Finkelstein S.E., Klebanoff C.A., Antony P.A., Palmer D.C., Spiess P.J., Hwang L.N., Yu Z., Wrzesinski C., Heimann D.M. (2005). Removal of homeostatic cytokine sinks by lymphodepletion enhances the efficacy of adoptively transferred tumor-specific CD8+ T cells. J. Exp. Med..

[48] Salem M.L., Díaz-Montero C.M., Al-Khami A.A., El-Naggar S.A., Naga O., Montero A.J., Khafagy A., Cole D.J. (2009). Recovery from cyclophosphamide-induced lymphopenia results in expansion of immature dendritic cells which can mediate enhanced prime-boost vaccination antitumor responses in vivo when stimulated with the TLR3 agonist poly(I:C). J. Immunol..

[49] Gershan J.A., Barr K.M., Weber J.J., Jing W., Johnson B.D. (2015). Immune modulating effects of cyclophosphamide and treatment with tumor lysate/CpG synergize to eliminate murine neuroblastoma. J. Immunother. Cancer.

[50] Jacoby E., Yang Y., Qin H., Chien C.D., Kochenderfer J.N., Fry T.J. (2016). Murine allogeneic CD19 CAR T cells harbor potent antileukemic activity but have the potential to mediate lethal GVHD. Blood.

[51] Derer A., Spiljar M., Bäumler M., Hecht M., Fietkau R., Frey B., Gaipl U.S. (2016). Chemoradiation Increases PD-L1 Expression in Certain Melanoma and Glioblastoma Cells. Front. Immunol..

