# Supplementary material for: Pre-Treatment with Dacarbazine Sensitizes B16 Melanoma to CAR T Cell Therapy in Syngeneic Mouse Model
Source: Int J Mol Sci. 2025 Dec 24;27(1):189. doi: 10.3390/ijms27010189 (PMC12785862; doi:10.3390/ijms27010189)
Supplement: Supplementary file 1 [file ijms-27-00189-s001.zip › ijms-3976117-supplementary.pdf]

Supplementary

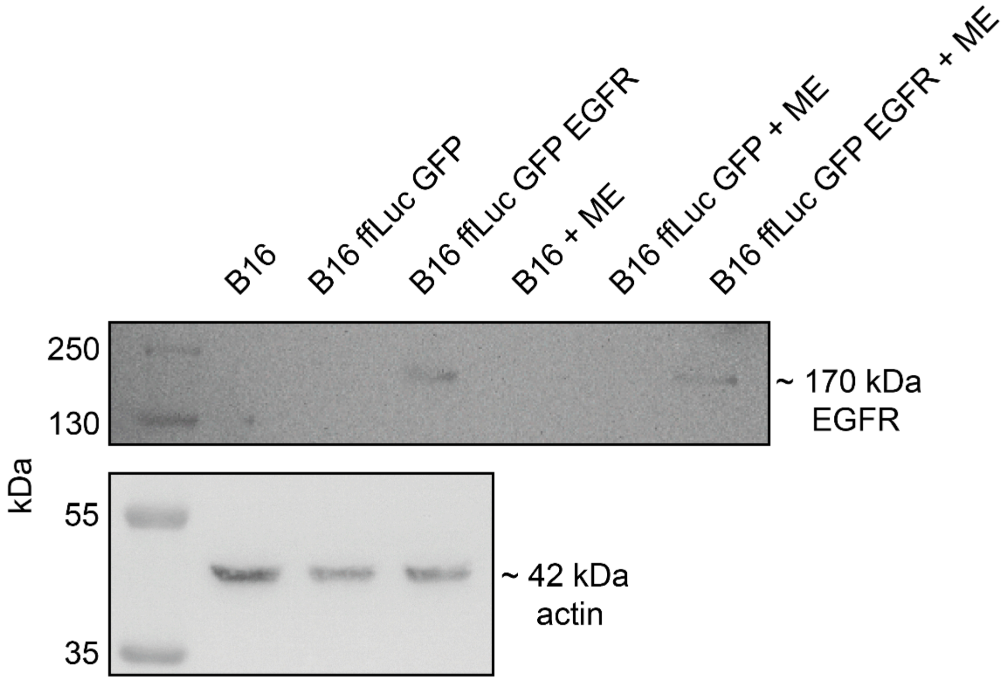

Figure S1. Western blotting of target cells

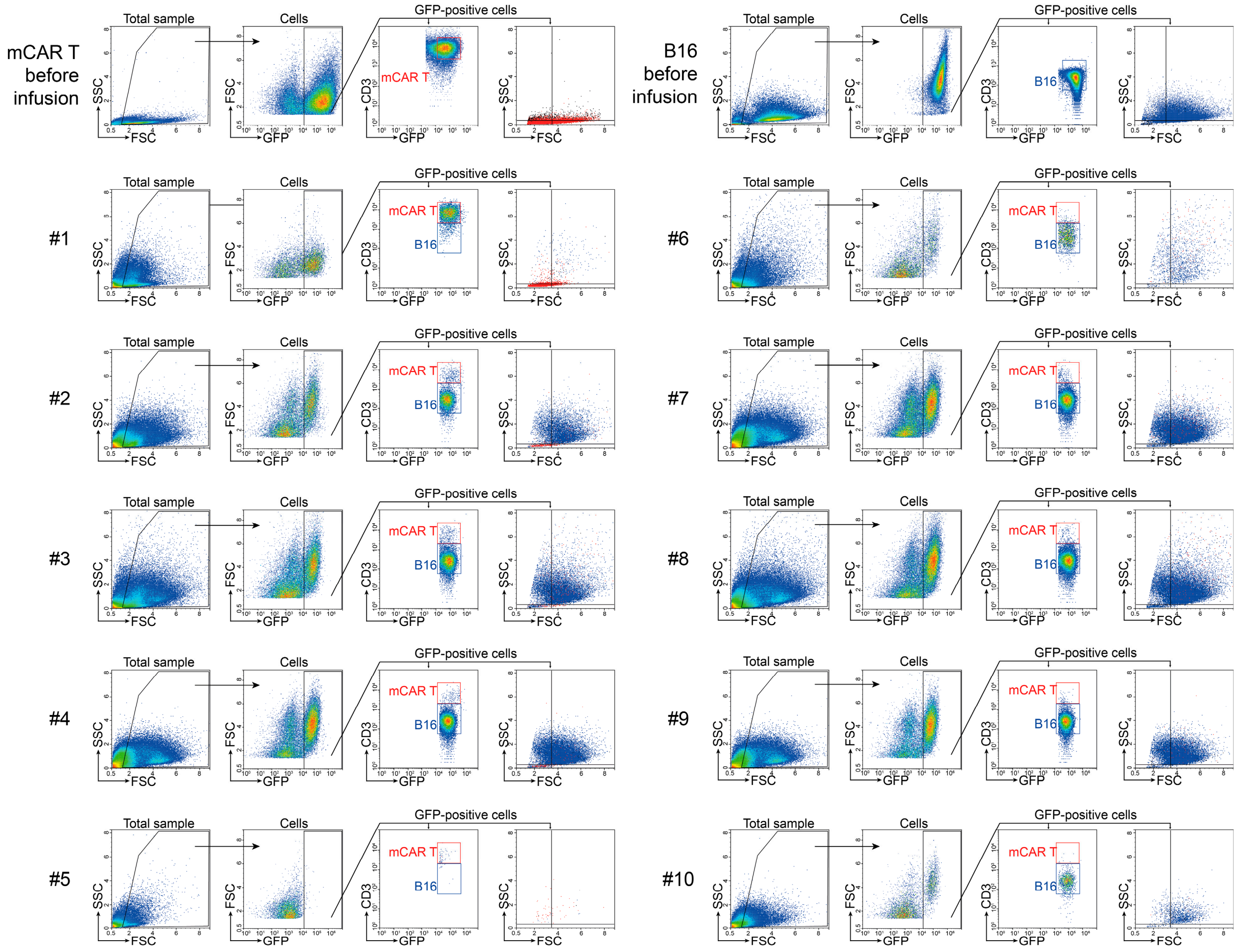

Figure S2. Flow Cytometry Gating for Tumor Samples (a key for group numbers #1-#10 can be found in Figure S3).

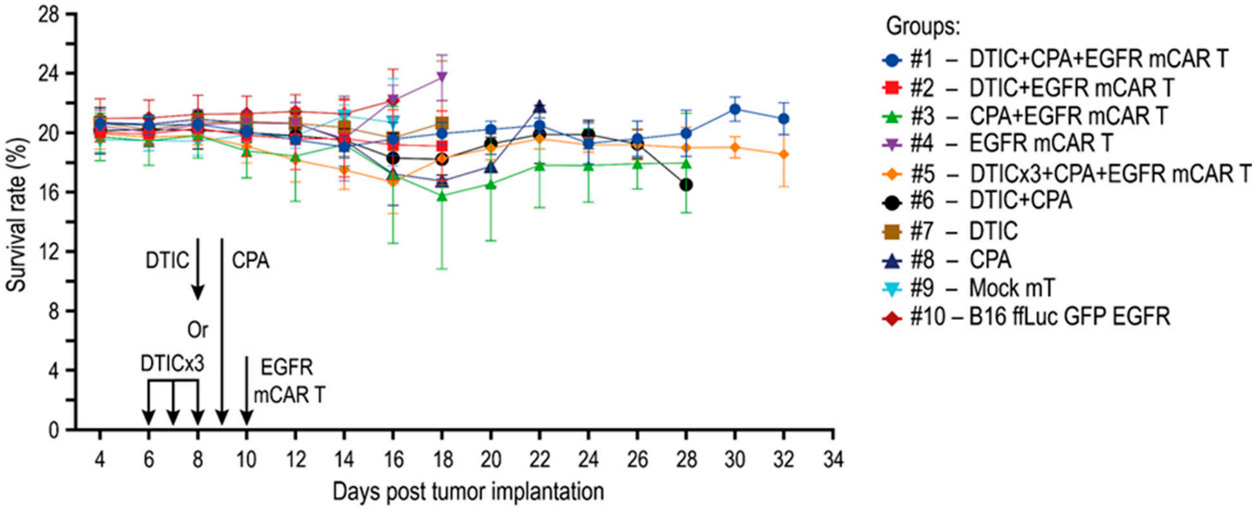

Figure S3. Body-weight dynamics during combinatorial treatment.

Table S1. Flow cytometry statistics for tumor samples (a key for group numbers #1-#10 can be found in Figure S3).

Group number	Total, absolute count	Cells, %	Cells, absolute count	GFP+ cells, %	GFP+ cells, absolute count	CD3+GFP+ cells (EGFR mCAR T cells), %	CD3+GFP+ cells (EGFR mCAR T cells), absolute count	CD3-GFP+ cells (B16 fLuc GFP EGFR cells), %	CD3-GFP+ cells (B16 fLuc GFP EGFR cells), absolute count
Group #1	109616 ± 5481	34,7500 ± 1,7375	38089 ± 1904	6,8300 ± 0,3415	7483 ± 374	5,4100 ± 0,2705	5933 ± 297	0,0500 ± 0,0025	50 ± 3
Group #2	131840 ± 6592	34,2400 ± 1,7120	45144 ± 2257	4,6300 ± 0,2315	6105 ± 305	0,2600 ± 0,0130	344 ± 17	3,3700 ± 0,1685	4441 ± 222
Group #3	165732 ± 8287	35,1500 ± 1,7575	58259 ± 2913	6,4500 ± 0,3225	10688 ± 534	0,1300 ± 0,0065	216 ± 11	4,9900 ± 0,2495	8268 ± 413
Group #4	144506 ± 7225	27,2400 ± 1,3620	39360 ± 1968	12,1900 ± 0,6095	17612 ± 881	0,1000 ± 0,0050	151 ± 8	10,0200 ± 0,5010	14485 ± 724
Group #5	62699 ± 3135	34,8000 ± 1,7400	21818 ± 1091	0,3600 ± 0,0180	227 ± 11	0,0300 ± 0,0015	18 ± 1	0,0000 ± 0,0000	2 ± 0
Group #6	111733 ± 5587	22,2600 ± 1,1130	24872 ± 1244	1,0100 ± 0,0505	1132 ± 57	0,0200 ± 0,0010	21 ± 1	0,6200 ± 0,0310	690 ± 35
Group #7	179268 ± 8963	43,8300 ± 2,1915	78577 ± 3929	10,6400 ± 0,5320	19078 ± 954	0,0500 ± 0,0025	97 ± 5	8,4900 ± 0,4245	15214 ± 761
Group #8	188015 ± 9401	26,5100 ± 1,3255	49839 ± 2492	10,2800 ± 0,5140	19319 ± 966	0,0500 ± 0,0025	89 ± 4	8,4700 ± 0,4235	15917 ± 796
Group #9	108603 ± 5430	29,8700 ± 1,4935	32442 ± 1622	13,1200 ± 0,6560	14250 ± 713	0,0600 ± 0,0030	61 ± 3	10,8200 ± 0,5410	11751 ± 588
Group #10	94159 ± 4708	21,8000 ± 1,0900	20525 ± 1026	1,6900 ± 0,0845	1588 ± 79	0,0100 ± 0,0005	11 ± 1	1,2800 ± 0,0640	1206 ± 60
